# Structure of *Arabidopsis* chloroplastic monothiol glutaredoxin AtGRXcp

**DOI:** 10.1107/S0907444910013119

**Published:** 2010-05-15

**Authors:** Lenong Li, Ninghui Cheng, Kendal D. Hirschi, Xiaoqiang Wang

**Affiliations:** a Plant Biology Division, Samuel Roberts Noble Foundation, 2510 Sam Noble Parkway, Ardmore, Oklahoma 73401, USA; b USDA/ARS Children’s Nutrition Research Center, Department of Pediatrics, Baylor College of Medicine, 1100 Bates Street, Houston, Texas 77030, USA

**Keywords:** monothiol glutaredoxins, AtGRXcp, *Arabidopsis thaliana*

## Abstract

Monothiol glutaredoxins (Grxs) play important roles in maintaining redox homeostasis in living cells and are con­served across species. *Arabidopsis thaliana* monothiol gluta­redoxin AtGRXcp is critical for protection from oxidative stress in chloroplasts. The crystal structure of AtGRXcp has been determined at 2.4 Å resolution. AtGRXcp has a glutaredoxin/thioredoxin-like fold with distinct structural features that differ from those of dithiol Grxs. The structure reveals that the putative active-site motif CGFS is well defined and is located on the molecular surface and that a long groove extends to both sides of the catalytic Cys97. Structural comparison and molecular modeling suggest that glutathione can bind in this groove and form extensive interactions with conserved charged residues including Lys89, Arg126 and Asp152. Further comparative studies reveal that a unique loop with five additional residues adjacent to the active-site motif may be a key structural feature of monothiol Grxs and may influence their function. This study provides the first structural information on plant CGFS-type monothiol Grxs, allowing a better understanding of the redox-regulation mechanism mediated by these plant Grxs.

## Abstract 2

The structure of *Arabidopsis* monothiol glutaredoxin AtGRXcp has been determined and reveals unique structural features of monothiol glutaredoxins, key residues for their interaction with glutathione and structural determinants for their distinct biochemical properties.

## 1. Introduction

Glutaredoxins (Grxs) are ubiquitous small heat-stable oxido­reductases that are conserved in both prokaryotes and eukaryotes (Lillig *et al.*, 2008 ▶). Grxs catalyze the reduction of protein disulfides and of glutathione (GSH)–protein mixed disulfides *via* a dithiol or monothiol mechanism (Bushweller *et al.*, 1992 ▶). The dithiol Grxs contain a conserved -Cys-*X*-*X*-Cys- active-site motif (Lillig *et al.*, 2008 ▶). In addition to this redox center, Grxs possess a binding site for glutathione, which is a ubiquitous tripeptide γ-Glu-Cys-Gly and the major biological thiol compound (Nikkola *et al.*, 1991 ▶). Recently, human mitochondrial Grx2 and poplar GrxC1 have been identified as iron–sulfur [2Fe–2S] cluster-containing proteins (Johansson *et al.*, 2007 ▶; Rouhier *et al.*, 2007 ▶; Lillig *et al.*, 2005 ▶; Feng *et al.*, 2006 ▶). This [2Fe–2S] cluster has been proposed to act as a redox sensor for activation of the Grx under stress conditions (Lillig *et al.*, 2005 ▶). These findings suggest that Grxs are important for regulating the redox state in living cells (Lillig *et al.*, 2008 ▶).

Recently, a new monothiol subgroup of Grxs has been identified (Herrero & de la Torre-Ruiz, 2007 ▶). Monothiol Grxs contain a single cysteine residue in the putative active-site motif C*XX*S (Herrero & de la Torre-Ruiz, 2007 ▶; Tripathi *et al.*, 2008 ▶; Izquierdo *et al.*, 2008 ▶; Mesecke, Spang *et al.*, 2008 ▶) and are conserved across species (Herrero & de la Torre-Ruiz, 2007 ▶). It has been shown that monothiol Grxs have diverse biological functions such as protection against protein oxidation in chloroplasts, biogenesis of iron–sulfur clusters in mitochondria and regulation of iron homeostasis (Herrero & de la Torre-Ruiz, 2007 ▶). However, biochemical studies have revealed that unlike dithiol Grxs, the majority of monothiol Grxs (*e.g.* CGFS-type Grxs) do not possess oxidoreductase activity even though these monothiol Grxs contain the con­served N-terminal cysteine residue (Herrero & de la Torre-Ruiz, 2007 ▶; Lillig *et al.*, 2008 ▶). Therefore, it is still unclear how and what structural determinants contribute to the biochemical properties of this group of Grxs.

Structures of a number of dithiol Grxs have been determined by X-ray and NMR, including poxviral Grx (Bacik & Hazes, 2007 ▶), bacterial Grx2 and Grx3 (Nordstrand *et al.*, 2000 ▶; Xia *et al.*, 2001 ▶; Foloppe *et al.*, 2001 ▶), yeast Grx1 (Håkansson & Winther, 2007 ▶), poplar GrxC1 (Feng *et al.*, 2006 ▶; Rouhier *et al.*, 2007 ▶), pig liver Grx (Katti *et al.*, 1995 ▶) and human Grx1 and Grx2 (Sun *et al.*, 1998 ▶; Johansson *et al.*, 2007 ▶). The glutathione-binding sites of human Grx2 (Johansson *et al.*, 2007 ▶) and bacterial Grx3 (Nordstrand *et al.*, 1999 ▶; Sheng *et al.*, 2007 ▶) have also been defined. Glutathione binds at the protein surface and its Cys forms a disulfide bond with the N-terminal cysteine of the active-site C*XX*C motif. Only a few structures of monothiol Grxs have been determined (Fladvad *et al.*, 2005 ▶; Gibson *et al.*, 2008 ▶; Iwema *et al.*, 2009 ▶). The structures of two monothiol Grxs, *Escherichia coli* Grx4 and the Trx-like domain of yeast Grx3, have been reported. However, the active-site motif regions are not visible or are partially dis­ordered in two of these monothiol Grx structures (Fladvad *et al.*, 2005 ▶; Gibson *et al.*, 2008 ▶). More recently, the structure of poplar GrxS12 has been determined (Couturier *et al.*, 2009 ▶). This enzyme possesses an unusual monothiol CSYS active-site sequence and is similar to yeast ScGrx6 which contains the CSYS motif (Mesecke, Mittler *et al.*, 2008 ▶; Couturier *et al.*, 2009 ▶). In contrast to some other monothiol Grxs, GrxS12 does not incorporate an iron–sulfur cluster in its original form, whereas *E. coli* Grx4 has been demonstrated to bind an iron–sulfur cluster in its homodimeric form (Iwema *et al.*, 2009 ▶; Couturier *et al.*, 2009 ▶). To date, no structure has been reported of a plant monothiol CGFS-type Grx.

*Arabidopsis* chloroplastic Grx, AtGRXcp, was the first plant monothiol Grx to be characterized and plays an important role in redox regulation and protection against oxidative stress in chloroplasts (Cheng *et al.*, 2006 ▶). It has also been shown that AtGRXcp is able to rescue the lysine auxotrophy of a yeast *grx5* mutant, suggesting that AtGRXcp may have a similar function in the maturation of the iron–sulfur cluster assembly (Cheng *et al.*, 2006 ▶; Herrero & de la Torre-Ruiz, 2007 ▶). Furthermore, biochemical studies have indicated that nine CGFS-type Grxs, including AtGrx5p (AtGRXcp), can bind a [2Fe–2S] cluster (Picciocchi *et al.*, 2007 ▶). However, the structural basis of the biochemical properties of AtGRXcp has not been defined. Here, we report the first crystal structure of the CGFS-type monothiol glutaredoxin AtGRXcp. The structure reveals distinct features that differ from those of dithiol Grxs. The structural analysis reveals a putative binding groove for glutathione. Structural comparative analysis shows that a glutathione molecule may fit into this groove, form a disulfide bond with the catalytic Cys97 and interact with several charged residues including Lys89, Arg126 and Asp152. Further comparative studies of structures and sequences reveal that monothiol Grxs have a unique loop with five additional residues adjacent to the active-site motif which may be a key structural determinant for their function.

## 2. Materials and methods

### 2.1. Cloning, protein expression and purification

AtGRXcp contains a 63-amino-acid signal peptide that targets the protein to chloroplasts (Cheng *et al.*, 2006 ▶). This N-­terminal signal peptide was removed and a truncated form of AtGRXcp (AtGRXcp63d) was amplified by PCR and cloned into the bacterial expression vector pET-41a (Novagen, Madison, Wisconsin, USA) as described previously (Cheng *et al.*, 2006 ▶). *E. coli* BL21 (DE3) cells harboring the expression construct were grown at 310 K in LB medium containing 50 µg ml^−1^ kanamycin. At an OD_600_ of 0.6–0.8, expression of proteins was induced by addition of isopropyl β-d-1-thio­galactopyranoside (IPTG) to a final concentration of 1 m*M*. After further incubation at 289 K overnight, the cells were pelleted and resuspended in lysis buffer (20 m*M* Tris–HCl pH 7.5, 1 *M* NaCl, 10 m*M* imidazole, 1 m*M* DTT) and homogenized with a French press; the complete lysates were centrifuged at 20 000*g* at 277 K for 40 min. The supernatant containing the His-tagged proteins was transferred onto a His GraviTrap column (GE Healthcare) and the column was washed extensively with lysis buffer (about 100 column volumes). The bound His-tagged proteins were eluted with elution buffer (20 m*M* Tris–HCl pH 7.5, 1.0 *M* NaCl, 250 m*M* imidazole, 1 m*M* DTT). The eluted proteins were cleaved with enterokinase to remove both GST and His tags and then dialyzed overnight at 277 K against dialysis buffer (20 m*M* Tris–HCl pH 7.5, 100 m*M* NaCl, 1 m*M* DTT). Dialyzed proteins were further purified on a Superdex-75 gel-filtration column (GE Healthcare) and concentrated to 6–10 mg ml^−1^ in 10 m*M* NaCl, 1 m*M* DTT, 20 m*M* Tris–HCl pH 7.0.

### 2.2. Crystallization and data collection

Crystallization of AtGRXcp protein was carried out using the hanging-drop vapor-diffusion method. The crystals were obtained from 10% 2-methyl-2,4-pentanediol (MPD), 1.0 *M* K_2_HPO_4_/NaH_2_PO_4_ pH 8.5. Crystals grew over 4 d to dimensions of ∼0.3 × 0.2 × 0.1 mm. Prior to data collection, the crystals were transferred to a cryo-solution containing 40% MPD with mother liquor and flash-cooled to 93 K. Data from a protein crystal were measured to 2.4 Å resolution using an R-­AXIS IV++ image-plate detector and RU-H3R rotating-anode X-ray source. All data were processed and scaled with the *HKL*-2000 software package (Otwinowski & Minor, 1997 ▶).

### 2.3. Structure determination and refinement

The structure of AtGRXcp was solved by molecular replacement using the program *Phaser* (Read, 2001 ▶) and the *E. coli* Grx4 structure (PDB code 1yka) as a search model (Fernandes *et al.*, 2005 ▶). Interactive model building and crystallographic refinement were carried out using the programs *Coot* (Emsley & Cowtan, 2004 ▶) and *CNS* (Brünger *et al.*, 1998 ▶), respectively. A bulk-solvent correction was applied. Restrained individual *B*-factor refinement was carried out. Water molecules were added using the *ARP*/*wARP* (Lamzin *et al.*, 2001 ▶) program and checked with an *F*_o_ − *F*_c_ map; 84 water molecules were included in the final model. The program *PROCHECK* (Laskowski *et al.*, 1993 ▶) was used to check the model. All backbone ϕ–ψ torsion angles were within allowed regions of the Ramachandran plot.

### 2.4. Molecular docking

Glutathione was docked into the AtGRXcp active site by superimposing the structures of Grxs bound with GSH on that of AtGRXcp. The structure of human Grx2 complexed with glutathione (PDB code 2fls; Johansson *et al.*, 2007 ▶) was used as a template. The dimer of AtGRXcp was generated by superimposing two AtGRXcp molecules onto the poplar GrxC1 dimeric structure bound with a [2Fe–2S] cluster (PDB code 2e7p; Rouhier *et al.*, 2007 ▶). The program *Coot* was used to adjust the models, to analyze the hydrogen bonds and van der Waals contacts between ligands and proteins and to optimize the binding mode.

## 3. Results and discussion

### 3.1. Overall structure

The crystal structure of *Arabidopsis* monothiol gluta­redoxin AtGRXcp was determined at 2.4 Å resolution by molecular replacement and refined to an *R* factor of 19.2% and an *R*_free_ of 22.6%. Data-collection and refinement statistics are presented in Table 1 ▶.

**Figure 1:**
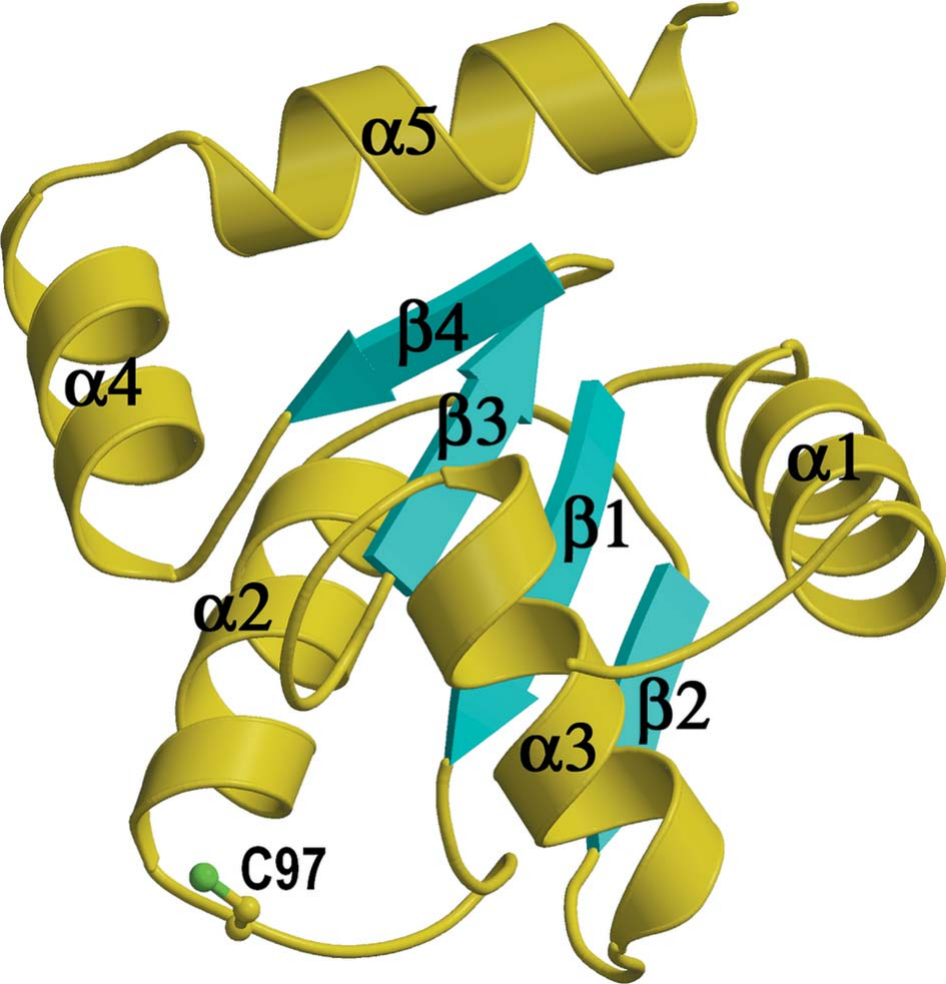
Ribbon diagram of the structure of AtGRXcp. The secondary structures are labeled. Figs. 1, 4, 5(*b*), 5(*c*) and 6 were prepared with *MolScript* (Kraulis, 1991 ▶; Couturier *et al.*, 2009 ▶) and *RASTER*3*D* (Merritt & Bacon, 1997 ▶).

The structure of AtGRXcp has a glutaredoxin/thioredoxin-like fold with a core four-stranded parallel β-sheet flanked by five α-helices on both sides (Figs. 1 ▶ and 2 ▶). AtGRXcp is classified as a monothiol glutaredoxin with a CGFS active-site motif. Its catalytic cysteine (Cys97) is between the β1 strand and α2 helix and is located on the molecular surface.

There is only one protein molecule in the crystallographic asymmetric unit. The structural model contains residues 65–173 of AtGRXcp, the chloroplastic signal peptide of which is removed (Cheng *et al.*, 2006 ▶). The electron-density map for the structure is well defined (Fig. 3 ▶).

### 3.2. Comparison with dithiol and monothiol Grxs

Several dithiol and monothiol Grx structures have been reported, including *E. coli* monothiol glutaredoxin Grx4 (PDB codes 1yka and 2wci; Fladvad *et al.*, 2005 ▶; Iwema *et al.*, 2009 ▶). Structural comparison reveals that AtGRXcp is highly similar to *E. coli* Grx4, with a root-mean-square deviation (r.m.s.d.) of 1.3 Å (2wci) or 1.8 Å (1yka) for 103 C^α^ atoms and a sequence identity of 36% (Fig. 4 ▶ *a*). The active site in the *E. coli* Grx4 structure is partially disordered (Fladvad *et al.*, 2005 ▶). In the AtGRXcp crystal structure the active site is well defined in the electron-density map (Fig. 3 ▶ *a*). Large differences are observed in five different loop regions, including the active-site motif region, with a distance of 8.5 Å between the C^α^ atoms of Arg92 of AtGRXcp and the corresponding residue Pro25 of *E. coli* Grx4.

The crystal structure of the N-terminal Trx-like domain of yeast monothiol Grx3 has been reported and its active-site motif region is disordered (PDB code 3d6i; Gibson *et al.*, 2008 ▶). Structural comparisons between AtGRXcp and Grx3 show very large differences, with an r.m.s.d. of 4.1 Å for 79 C^α^ atoms and a sequence identity of 13%. The α1 helix of Grx3 is in a different location and thus could not be superimposed on the corresponding region of AtGRXcp. The active-site motif of Grx3 is defined in one of the two molecules in the asymmetric unit and the catalytic Cys72 is located in the opposite direction compared with the AtGRXcp structure. These comparative studies indicate that the active-site motifs in monothiol Grxs are likely to be flexible and some conformational changes may occur when a ligand binds to an enzyme.

Comparison of AtGRXcp and the recently reported structure of poplar GrxS12 (Couturier *et al.*, 2009 ▶) gives an r.m.s.d. of 1.3 Å for 99 C^α^ atoms and 30% sequence identity. GrxS12 has an unusual monothiol CSYS active-site motif instead of a CGFS motif (Couturier *et al.*, 2009 ▶). Recent studies have revealed that GrxS12 from poplar, PfGLP2 (CKFS motif) and PfGLP3 (CKYS motif) from *Plasmodium falciparum*, ScGrx6 (CSYS motif) and ScGrx7 (CPYS motif) from yeast and 1-C-Grx1 (CAYS motif), 1-C-Grx2 (CGFT motif) and 1-­C-Grx3 (CGFT motif) from *Trypanosoma brucei* do not contain the CGFS motif (Deponte *et al.*, 2005 ▶; Mesecke, Mittler *et al.*, 2008 ▶; Filser *et al.*, 2008 ▶). In contrast to most monothiol Grxs, yeast ScGrx6 and ScGrx7 and poplar GrxS12 have GSH-dependent oxidoreductase activity like dithiol Grxs (Mesecke, Mittler *et al.*, 2008 ▶; Couturier *et al.*, 2009 ▶). Together, these findings imply that additional structural determinants are required for the function of monothiol Grxs.

Structural comparison also reveals a high similarity between AtGRXcp and the classic dithiol Grxs (Fig. 4 ▶ *b*), including poplar GrxC1 (PDB code 2e7p; r.m.s.d. of 1.8 Å for 102 C^α^ atoms, 29% sequence identity; Rouhier *et al.*, 2007 ▶), yeast Grx2 (PDB code 3d4m; r.m.s.d. of 1.5 Å for 102 C^α^ atoms, 24% sequence identity; Discola *et al.*, 2009 ▶), human Grx2 (PDB code 2fls; r.m.s.d. of 1.4 Å for 98 C^α^ atoms, 20% sequence identity; Johansson *et al.*, 2007 ▶), *E. coli* Grx3 (PDB code 3grx; r.m.s.d. of 1.7 Å for 81 C^α^ atoms, 25% sequence identity; Nordstrand *et al.*, 1999 ▶) and poxviral Grx (PDB code 2hze, r.m.s.d. of 2.7 Å for 99 C^α^ atoms, 21% sequence identity; Bacik & Hazes, 2007 ▶), although the sequence identities are low. The largest differences between AtGRXcp and these dithiol Grxs are also observed in the active-site regions of these enzymes.

The average temperature factor of the active-site motif region is 47 Å^2^ for AtGRXcp, which is slightly lower than the overall average value of 49 Å^2^. The average temperature factors of the corresponding regions are 9 Å^2^ (the overall value is 35 Å^2^) for poplar GrxC1 (Rouhier *et al.*, 2007 ▶), 6.5 Å^2^ (overall value 16.2 Å^2^) for yeast Grx1 (PDB code 3c1r; Yu *et al.*, 2008 ▶), 6.4 Å^2^ (overall value 17.1 Å^2^) for reduced Grx2 (PDB code 3ctg; Li *et al.*, 2010 ▶) and 7.4 Å^2^ (overall value 27.6 Å^2^) for oxidized Grx2 (PDB code 3ctf; Li *et al.*, 2010 ▶), which are much lower than the overall values. This suggests that the conformation of the active-site motif in AtGRXcp is less stable than that in dithiol Grxs, which is consistent with our earlier conclusion that the active-site motif in monothiol Grxs is more flexible.

### 3.3. The binding groove for glutathione

**Figure 2:**
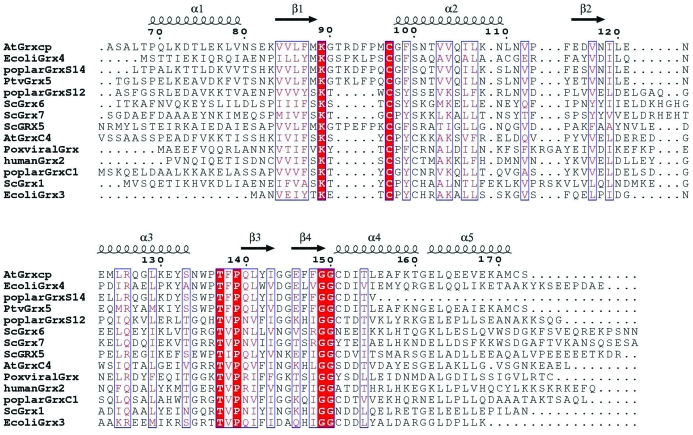
Structure-based sequence alignment of monothiol and dithiol Grxs, including AtGRXcp, AtGrxC4, *E. coli* Grx3 and Grx4, poplar GrxC1 and GrxS14, *Pteris vittata* Grx5, yeast Grx1, Grx5, Grx6 and Grx7, poxviral Grx and human Grx2. This figure was produced using *ENDscript* (Gouet & Courcelle, 2002 ▶).

In the structure of AtGRXcp, the catalytic Cys97 is solvent-exposed (Fig. 1 ▶). A long groove is observed adjacent to Cys97 with a width of 11–14 Å and a length of 16–19 Å (Fig. 5 ▶). The groove is formed by highly conserved residues present in plant monothiol Grxs (Fig. 2 ▶) and would be the binding site for glutathione (GSH), *i.e.* a γ-­Glu-Cys-Gly tripeptide.

Molecular docking and comparison with the structure of human Grx2 complexed with glutathione (PDB code 2fls; Johansson *et al.*, 2007 ▶) show that in the structure of AtGRXcp the glutathione (GSH) could fit the binding groove well and formed similar inter­actions between GSH and AtGRXcp (Fig. 5 ▶ *c*). The glycine of GSH is surrounded by positively charged residues (Arg126, Lys89 and Lys130) in AtGRXcp. In human Grx2, Lys34 and Gln69 interact with the carboxylates of the glycine of GSH (Johansson *et al.*, 2007 ▶). In AtGRXcp, Lys89 and Arg126 in corresponding positions might form salt-bridge interactions with the glycine residue in GSH. Lys130 is also close to the GSH glycine. A hydrogen-bonding network might be formed between Arg126, Lys130, Lys89 and the glycine of GSH, which anchor the C-terminus of the GSH.

The cysteine of GSH forms a disulfide bond with the catalytic cysteine and also interacts with the main-chain N and O atoms of Val81 in human Grx2 (Johansson *et al.*, 2007 ▶). Similarly, in the structure of AtGRXcp the active-site Cys97 forms a disulfide bond with the cysteine of GSH and the main-chain N and O atoms of Phe138 form hydrogen-bond interactions with the main-chain O and N atoms of the GSH cysteine.

The GSH glutamate interacts with the main-chain N atoms of Ala94 and Thr95 and the side chain of Thr95 in the human Grx2 structure (Johansson *et al.*, 2007 ▶). The corresponding residues in AtGRXcp are Cys151 and Asp152 and their backbone atoms are located in similar positions and could also form similar interactions with GSH; the Asp152 side chain would also be involved in interactions with GSH. Asp152 is the only negatively charged residue in the groove and is a conserved residue in monothiol Grxs. The side chain of Phe99 is close to the backbone of the glutamate of GSH and may enable a hydrophobic interaction. Trp135 is nearby and might interact with the carboxylate of the GSH glutamate. These observations suggest that the negatively charged environment provided by Asp152 and the hydrophobic interactions caused by Phe99 play a role in stabilizing the N-terminus of the GSH. Interestingly, a previous study indicated that the Phe99Ala mutant was capable of complementing the yeast *grx5* mutant function, while protein expression of the Cys97Ala mutant was affected (Cheng *et al.*, 2006 ▶). This observation could be explained by the fact that the substitution of Phe99 by Ala in AtGRXcp reduces the size of the side chains, but may not affect the binding of glutathione and the catalytic activity of monothiol gluta­redoxin. This is also consistent with the results from our crystallization experiments, in which crystals were obtained for the protein with the single amino-acid mutation Phe99Ala, but not with Cys97Ala (data not shown).

Under the crystallization conditions, we were unable to obtain crystals of the AtGRXcp–GSH complex by adding GSH to the crystallization solution. Structural analysis of AtGRXcp shows that Asp152 of a symmetry-related AtGRXcp occupies a portion of the GSH-binding groove and might prevent a GSH molecule from directly binding to the groove.

In addition, comparative structural studies and sequence-alignment analysis of monothiol and dithiol Grxs reveal that monothiol Grxs (*e.g.* CGFS-type Grxs), with the exceptions of ScGrx6, ScGrx7 and GrxS12, have five additional amino acids (*i.e.* Thr91-Arg92-Asp93-Phe94-Pro95 in AtGRXcp) immediately upstream of the active-site Cys97 (Figs. 2 ▶ and 4 ▶). Most interestingly, similar to dithiol Grxs, ScGrx6, ScGrx7 and GrxS12 lack these five amino-acid residues (Fig. 2 ▶) and are also active in hydroxyethyl disulfide HEDS assays and have GSH-dependent oxido­reductase activity (Mesecke, Spang *et al.*, 2008 ▶; Mesecke, Mittler *et al.*, 2008 ▶). This long unique loop with five additional residues adjacent to the catalytic Cys97 may be a key structural feature of monothiol Grxs.

### 3.4. Model of the Fe–S cluster

Both CGFS-type monothiol Grxs (*e.g.* SyGrx3p) and dithiol Grxs (*e.g.* poplar GrxC1 with an active-site sequence CGYC) may exist as a dimeric iron–sulfur cluster-containing holo­protein (Picciocchi *et al.*, 2007 ▶; Bandyopadhyay *et al.*, 2008 ▶; Rouhier *et al.*, 2007 ▶). The structural study shows that poplar GrxC1 is organized as a tetramer containing one [2Fe–2S] cluster that probably results from cocrystallization of the holo and apo forms (Rouhier *et al.*, 2007 ▶). However, the dimeric structure bound with a [2Fe–2S] cluster is likely to provide a good representation of the holodimer in solution and the [2Fe–2S] cluster is surrounded by the active-site motif and GSH.

The presence of a proline residue adjacent to the catalytic cysteine in poplar GrxC2, GrxC3 and GrxC4 is proposed to interfere with cluster formation and the presence of a small residue, especially a glycine, is likely to be essential for [2Fe–2S] cluster incorporation. Similarly, yeast ScGrx6 with a serine in the CSYS motif binds the [2Fe–2S] cluster, but ScGrx7 with a proline (CPYS motif) does not (Mesecke, Mittler *et al.*, 2008 ▶). AtGRXcp contains a glycine at the corresponding position and therefore should allow the incorporation of a [2Fe–2S] cluster. In agreement with this, a previous study demonstrated that AtGrx5p (AtGRXcp) can bind a [2Fe–2S] cluster (Picciocchi *et al.*, 2007 ▶). We speculate that AtGRXcp may form a similar dimer as poplar GrxC1 when binding to a [2Fe–2S] cluster. The [2Fe–2S] cluster might interact with the side chains of Cys97 and Phe99 and the main chain of the active-site motif as well with the GSH cysteine side chain (Fig. 6 ▶). Thus, the incorporation of a GSH-ligated [2Fe–2S] center is a common feature of both monothiol and dithiol Grxs.

### 3.5. Intermolecular disulfide-bond interaction

**Figure 3:**
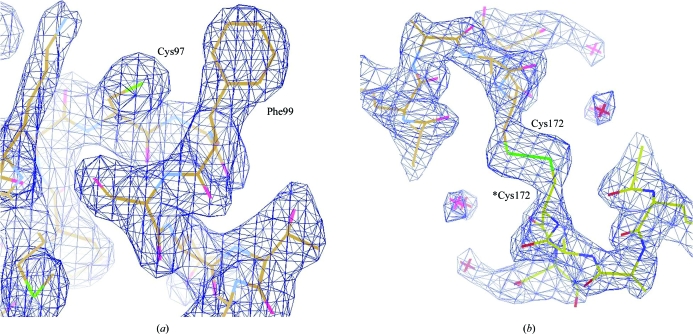
2|*F*_obs_| − |*F*_calc_| electron-density map contoured at 1.5σ of (*a*) the active-site motif region and (*b*) the intermolecular disulfide bond formed between Cys172 and *Cys172 of a symmetry-related Grx molecule.

**Figure 4:**
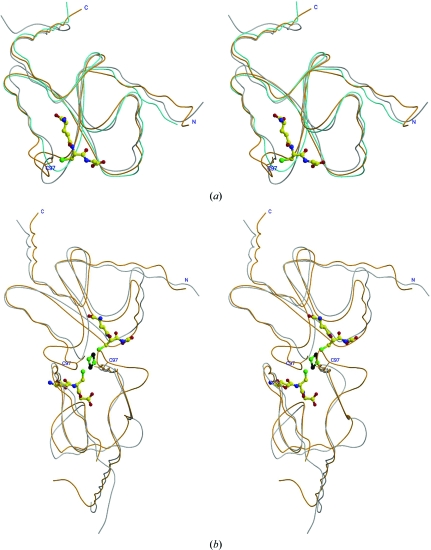
Stereo diagram showing the superimposition of the structures of AtGRXcp (orange) with (*a*) *E. coli* Grx4 (grey; PDB code 1yka) and human Grx2 (cyan; PDB ID 2fls) or (*b*) poplar GrxC1 dimer (grey; PDB code 2e7p) in which two AtGRXcp molecules are superimposed on the GrxC1 dimer. The GSH in human Grx2 and GSH and the [2Fe–2S] cluster in poplar GrxC1 are shown as ball-and-stick models.

**Figure 5:**
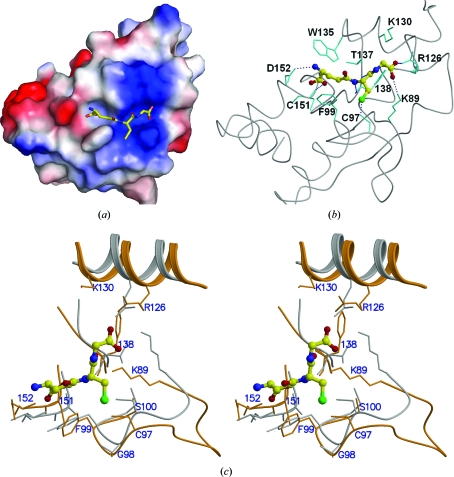
The putative glutathione-binding groove. (*a*) Electrostatic surface of the AtGRXcp with a docked GSH molecule. (*b*) Interaction between GSH and key amino-acid residues in the putative binding groove. (*c*) Stereo diagram showing the superimposition of the GSH-binding sites of AtGRXcp (orange) and human Grx2 (grey). The GSH in human Grx2 is shown as a ball-and-stick model.

**Figure 6:**
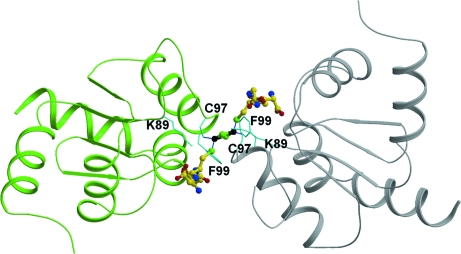
A putative dimer of AtGRXcp. The GSH molecules and [2Fe–2S] cluster were docked and are shown as ball-and-stick models. Some amino-acid residues are labeled and shown in cyan as bond models.

AtGRXcp possesses multiple cysteine residues including the active-site Cys97 and three other cysteines (Cys62, Cys151 and Cys172). Cys151 is conserved in most monothiol Grxs; it is located at the α4-helix and close to the glutathione-binding groove. Cys62 is within the chloroplast-targeting signal pep­tide and is not present in most CGFS-type Grxs; the corresponding Cys residue in PvGrx5 is involved in arsenic tolerance in brake fern (Sundaram *et al.*, 2008 ▶). The Cys172 residue in AtGRXcp is also not conserved in the monothiol Grxs. Interestingly, structural analysis showed that Cys172 is located in the α5-helix in the C-terminus on the molecular surface and forms an intermolecular disulfide bond with *Cys172 of a symmetry-related Grx molecule (Fig. 3 ▶ *b*). These two Grx molecules are related by a twofold crystallographic axis which is perpendicular to the threefold *c* axis. This interaction enhances the intermolecular interaction dramatically and the crystals possess high diffraction quality despite having a very high solvent content of 71.2%. This may be the driving force for the formation of such a crystal lattice under the crystallization conditions.

In this dimer structure, this disulfide bond Cys172–*Cys172 is the only interaction between the two Grx molecules, suggesting that AtGRXcp may aggregate by forming an intermolecular disulfide bond.

## 4. Conclusions

The overall structure of *Arabidopsis* monothiol glutaredoxin AtGRXcp is similar to those of dithiol and other monothiol Grxs, but there are unique features within the AtGRXcp structure that could determine the distinct biochemical properties displayed by the CGFS-type Grxs. Our structural findings strongly suggest that a long loop with five additional residues adjacent to the active-site motif may be a key structural feature of monothiol Grxs. It will be interesting to determine how this five-amino-acid stretch influences the function of this group of Grxs.

## Supplementary Material

PDB reference: AtGRXcp, 3ipz

## Figures and Tables

**Table 1:** Summary of data-collection and refinement statistics for AtGRXcp

Data statistics	
Space group	*P*321
Unit-cell parameters (Å, °)	*a* = 81.4, *b* = 81.4, *c* = 55.4, γ = 120
Resolution (Å)	2.4
Unique reflections	8608 (833)
Completeness (%)	99.8 (100)
*R*_merge_ (%)	6.7 (41.0)
〈*I*/σ(*I*)〉	21.4 (3.9)
Matthews coefficient (Å^3^ Da^−1^)	4.2
Solvent content (%)	71.2
Protein molecules in asymmetric unit	1
Refinement statistics	
*R* factor (%)	19.2
*R*_free_ (%)	22.6
No. of protein atoms	865
No. of water molecules	84
Average *B* factors (Å^2^)	45.6
R.m.s.d. from ideal values	
Bond lengths (Å)	0.008
Bond angles (°)	1.3
